# A commentary on “how to interpret expert judgment assessments of twenty-first century sea-level rise” by Hylke de Vries and Roderik SW van de Wal

**DOI:** 10.1007/s10584-016-1672-7

**Published:** 2016-06-07

**Authors:** JL Bamber, WP Aspinall, RM Cooke

**Affiliations:** 1 School of Geographical Sciences and Cabot Institute, University of Bristol, Bristol, BS8 1SS UK; 2 School of Earth Sciences and Cabot Institute, University of Bristol, Bristol, BS8 1RJ UK; 3 Department of Mathematics, TU Delft, The Netherlands (ret) and Resources for the Future, Washington, DC USA

**Keywords:** Statistical Accuracy, Expert Judgment, Expert Elicitation, Global Temperature Rise, Calibration Variable

## Abstract

We clarify key aspects of the evaluation, by de Vries and van de Wal (2015), of our expert elicitation paper on the contributions of ice sheet melting to sea level rise due to future global temperature rise scenarios (Bamber and Aspinall 2013), and extend the conversation with further analysis of their proposed approach for combining expert uncertainty judgments.

We thank de Vries and van de Wal (2015: [VW15]) for their detailed consideration of Bamber and Aspinall (2013: [BA13]), and welcome this opportunity to clarify the work presented in BA13 and extend the analysis of VW15. The problem of finding a science-based quantification of uncertainty for poorly constrained physical models with large societal impacts deserves high priority in the climate community. This entails crossing discipline boundaries and will take that community outside its usual scientific comfort zone. We therefore salute the authors of VW13 for venturing into this alien terrain and welcome the opportunity to address some of the issues they raise.

The present commentary discusses certain important and unique attributes of BA13’s expert weighting scheme that are misinterpreted in VW15, then addresses the “consensus distribution” of VW15, their “level of consensus”, and the issue of lognormal fitting elicited data.

## Weighting scheme

VW15 state: “*The answers of the experts* [in BA13] *were weighted using a specialised weighting technique*, *which involved the self-estimated level of expertise and confidence of the respondents*”.

This is not accurate: while it is correct that self-assessments were included in the original panel questionnaire, these responses were not used in the BA13 analysis. Instead, we used the Classical Model to weight experts individually.

With the Classical Model, experts’ performances in quantifying uncertainty are measured by eliciting uncertainties on calibration variables from their field whose values are known to them post hoc. Their responses are scored for statistical accuracy (measured as the *p*-value at which one would falsely reject the hypotheses that the probability assessments were statistically accurate), and for informativeness (measured as Shannon relative information with respect to a user supplied background measure), then a combined score is determined (the product of the two). Shannon relative information is used because it is scale invariant, tail insensitive, slow and familiar. The combined score satisfies a long run proper scoring rule constraint, and involves choosing an optimal statistical accuracy threshold beneath which experts are unweighted (Cooke 1991). A mathematically complete exposition of the Classical Model, in- and out-of-sample validation, extensive review of literature and case studies are available online.^1^ An extensive review of pre-2006 Classical Model case history data is Cooke and Goossens (2008), with further expositions in Cooke et al. (2014) and Cooke (2015, with supplementary online information).

Thus, at the core of VW15’s analysis of BA13 there appears to be a misunderstanding of these basic scoring principles. While we welcome discussion of this topic and acknowledge that there are alternative methods for combining expert judgments, it is essential, when critically assessing BA13, to understand precisely what has been done, and why. We may be unable to validate our models with physical measurements, but we can validate the experts who assess the uncertainties.

## Consensus distribution

When analysing data from BA13, VW15 construct what they term a ‘consensus distribution’ as follows: “*We determine a* “*consensus*” *distribution for each ice sheet and for each quantile* (*LO*, *MED and HI*) *by fitting a polynomial of degree p through the quantile-quantile plot*:

$$ y\sim {\varSigma}_{j=0\dots p}{a}_j{x}^j+\varepsilon $$

*with noise*, *ε*, *y the quantiles of the raw data and x the quantiles of a normal distribution. We take p* = *3 to account for the skewness in the expert opinions.*” (p.94). The authors do not mention how exactly the fitting was done. If they minimized sum squared error, per question, per elicited quantile, then the solution will always be the simple arithmetic average of the experts’ quantiles. Indeed:

$$ argmi{n}_z\kern0.5em {\varSigma}_{i=1\dots n}{\left({y}_i\hbox{--} z\right)}^2=\left(1/n\right){\varSigma}_{i=1\dots n}{y}_i $$

If we replace *z* by some function, say *f* (*a*_*0*_,…*a*_*3*_) = *Σ*_*j*=*0*…*p*_ *a*_*j*_ *x* ^*j*^, and choose (*a*_*0*_,…*a*_*3*_) to minimize the sum squared error, then the result will always be the average of the *y*_*i*_’s, provided this average is in the orbit of *f*(*a*_*0*_,…*a*_*3*_)*.* That is certainly the case here, as we can always put:

$$ {a}_0=\kern0.5em \left(1/n\right){\varSigma}_{i=1\dots n}{y}_i,{a}_1={a}_2={a}_3=0 $$

Of course there will be other values for (*a*_*0*_,…*a*_*3*_) which return the average, and the one any solver finds will depend on the starting point.

Thus, the consensus distribution is simply an unorthodox way of averaging the experts’ quantiles. Averaging quantiles is a common students’ mistake when trying to compute the average of experts’ distributions, but it has also been promoted in its own right (Lichtendahl et al. 2013), without checking its performance on real expert data. It appears to have eluded many that averaging quantiles is equivalent to harmonically weighting the experts’ densities. Let *F* and *G* be cumulative distribution functions (CDFs) from experts 1 and 2, with densities *f*, *g*. Let *HW*, *hw* denote respectively the CDF and density of the result of averaging the quantiles of *F*, *G*. Then:

1 $$ H{W}^{-1}(r)=1/2\left({F}^{-1}(r)+{G}^{-1}(r)\right) $$

Take derivatives of both sides:

2 $$ 1/hw\left(H{W}^{-1}(r)\right)=1/2\left(1/f\left({F}^{-1}(r)\right)+1/g\left({G}^{-1}(r)\right)\right) $$

3 $$ hw\left(H{W}^{-1}(r)\right)=\frac{2}{\left(1/f\left({F}^{-1}(r)\right)+1/g\left({G}^{-1}(r)\right)\right)} $$

Eq. (3) says that *hw* is the harmonic mean of *f* and *g*, evaluated at points corresponding to the *r* ^th^ quantile of each distribution. The harmonic mean of *n* numbers strongly favours the smallest of these numbers: the harmonic mean of 0.01 and 0.99 is 0.0198. This means that all regions of a distribution where one expert gives a low support density tend to get low densities, regardless what other experts say.

**Fig. 1:**
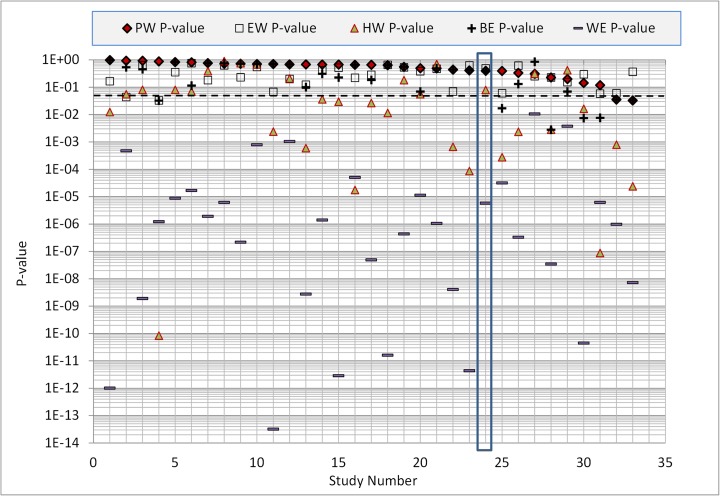
Statistical accuracy of performance weighting (*PW*), equal weighting (*EW*) harmonic weighting (*HW*, consensus distribution), the best expert (*BE*) and the worst expert (*WE*). Based on the calibration variables, these are the *p*-values of falsely rejecting the hypothesis that the assessments in question are statistically accurate. The best score is 1, the worst score is zero. Studies are ordered left-to-right according to statistical accuracy; BA13 is number 24 in this series

It is not surprising that the statistical accuracy of *HW* is poor. Thirty-three professional SEJ studies (including BA13) have been conducted since the data summary in Cooke and Goossens (2008). The analysis of these studies is on-going but interim results and all SEJ data can be found online here 2 (excluding data from a continuing application at the Montserrat Volcano Observatory, and a very recent application on the global burden of foodborne disease for WHO). Figure 1 summarizes the statistical accuracy (*p*-value, larger scores are better) of PW, EW, HW, the best and worst experts (BE, WE) in each panel. The results are ordered according to PW, BA13 is study nr. 24 (highlighted). The HW (consensus distribution), considered as a statistical hypothesis, would be rejected in 18 of the 33 panels at the traditional 5 % level, shown as dotted line in Fig. 1. In nine cases it would be rejected at the 0.1 % level. The wide spread between the statistical accuracy of PW, EW, HW, BE, and WE - with many values below the traditional rejection threshold - argues against applications of expert judgment without validation.

**Fig. 2:**
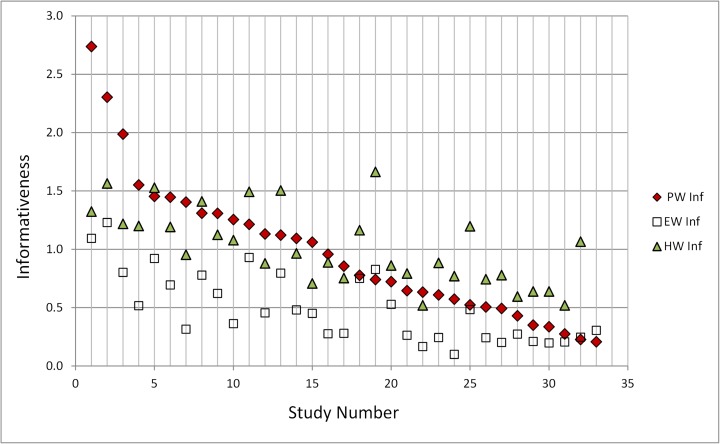
Informativeness of *PW*, *EW* and *HW*, ordered according to *PW* informativeness, left-to-right. Higher scores are better (more informative) as measured by Shannon relative information with respect to a common background measure. On this plot, BA13 is ranked number 4, and has a better informativeness score than its corresponding HW solution; in fact, only one elicitation of the other thirty-two cases has *HW* informativeness that is (marginally) better than BA13

Statistical accuracy does not tell the whole story. Informativeness is also important, though less important than statistical accuracy. Informative answers that are statistically very inaccurate are not useful, but given two solutions with good accuracy we should prefer the more informative. Figure 2, now ordered according to PW informativeness, shows that informativeness of harmonic weighting (HW) is usually comparable to that of PW, but here, for the BA13 case (study nr. 4), HW informativeness is lower than for the Classical Model PW solution.

## Level of consensus

**Fig. 3:**
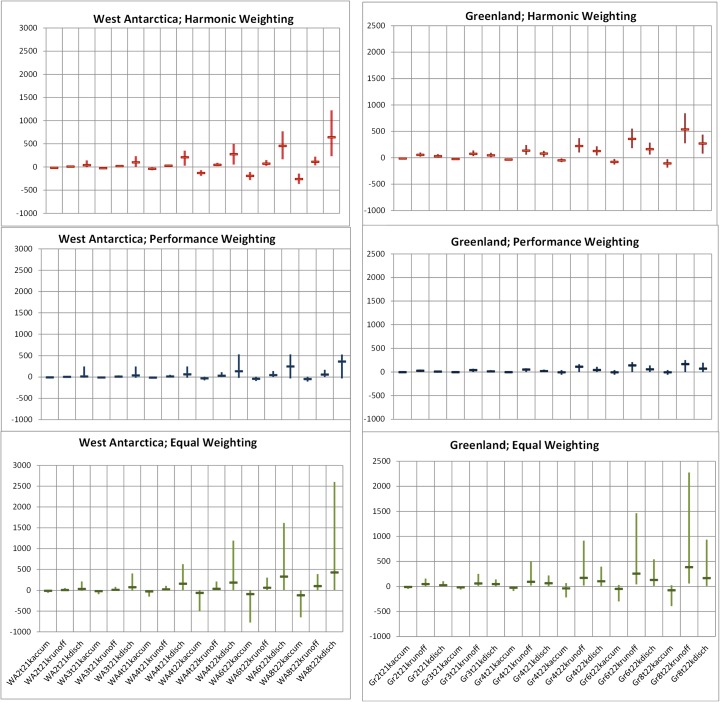
Harmonic, Performance and Equal weighting solutions for Greenland and West Antarctica ice sheet variables; y-axes indicate 90 % credible ranges for process-related sea level rise contributions under different warming scenarios, in mm, with median markers (see footnote 2 for explanation of variable codes)

In their Abstract, VW15 suggest that they are “*employing a method that keeps the level of consensus included*”*.* “Level of consensus” is repeatedly invoked in VW15, but not defined. Figure 3 shows the 90 % confidence bands and medians for harmonic weighting (consensus distribution), performance weighting and equal weighting, for all eighteen variables for the Greenland and West Antarctica ice sheets considered by the expert panel in BA13.^3^ Credible ranges are systematically wider for the higher temperature rise scenarios, and for projections to 2200 CE compared with those to 2100 CE.

The equal weight combinations (Fig. 3, bottom panels) reflect the heterogeneity in the experts’ judgments, and is typical for such elicitations (see e.g. Cooke and Goossens 2008 for a summary of such findings from elicitations to 2006).

Whereas equal weighting represents the views of all experts equally, performance weighting emphasizes the judgments of informative experts whose statistical accuracy is validated on calibration variables.

Absent a definition of “level of consensus” it is unclear what harmonic weighting (Fig. 3 upper) aims to achieve, and whether it has succeeded. Moreover, it is difficult to see how the consensus distribution is robust to the outliers reflected in the equal weight combinations. In any case, the statistical accuracy of harmonic weighting is quite poor, as shown in Fig. 1.

It is also unclear why a “consensus” approach is justified. In the present context, an analogy is the performance of coupled atmosphere-ocean Global Circulation Models (GCM). It has been known and accepted for many years that some GCMs have greater skill than others in, for example, hindcasting the temperature field of the Arctic (where model skill is a concept similar to, but not identical with, performance weighted solutions in the Classical Model). The climate modelling community has, therefore, accepted that weighting model ensembles (e.g. Murphy et al. 2004) or excluding low skill models results in better predictive performance.

## Lognormal fitting

VW15, at Section 2.2 p90, consider what type of distribution best fits the quantile data in BA13, and embark on a discussion of technical aspects of this task in relation to the use of the lognormal and its parameterization (i.e. determining the shape, scale and location parameters of that distribution).

In BA13, the spreads of the expert’s judgments on every item were elicited as three-point marker quantiles (5th, 50th and 95th percentiles) and these comprise the data that went into the Classical Model processing. If the experts’ initial uncertainty spreads evince some form of gross systematic discrepancy (e.g. “two schools of thought”, with non-overlapping uncertainty ranges), then this lack of consensus may be diagnostic of a deeper ambiguity, either in the target question or in the knowledge or experiences of the experts, and this would normally be the trigger to re-visit the issue with the experts. Such was not the case.

The BA13 elicitation results, when processed with EXCALIBUR, were expressed in terms of values for these three percentiles also, and were sensitive principally to the quantile values given by the experts. The default intrinsic range (Cooke 1991) facility of EXCALIBUR was used to define lower and upper bounds to the group-wide spread of values for each item and, for Monte Carlo re-sampling, we fitted special lognormal distributions using the RiskModel (Professional v4.3: Vose Software 2013) *LognormalAlt* formulation, which can accommodate negative and positive values. This fitting method did not require external decisions about the location parameter, as is the case of the τ-value in VW15.

We acknowledge that other choices for fitting distributions could be made, and it would be useful to revisit this issue once the more fundamental issues surrounding the “consensus distribution” are resolved.

## Conclusions

The stimulating contribution of VW15 provides a welcome opportunity to extend the discussion of some important aspects of expert elicitation for estimating ice sheet contribution to sea level rise, beyond what was possible in BA13. In applying the Classical Model, we sought a more reliable and reasoned way of enumerating poorly constrained uncertainties with structured expert judgment, and this was the rationale behind the BA13 approach. Justification for this choice is based on an extensive database of forty-five professional elicitation studies, each involving calibration variables and the Classical Model, published in Cooke and Goossens (2008).

**Fig. 4:**
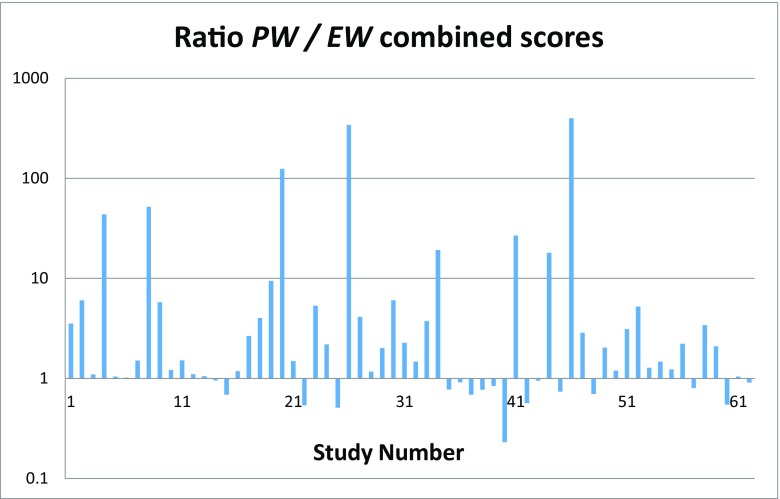
Eggstaff et al. (2014) results of cross validation ratios of combined scores *PW* / *EW* for 62 expert elicitation studies aggregated over all training / test set splits (reproduced from Cooke 2015)

Several researchers have used this database to study validation of expert judgment (for a review see Cooke et al. 2014). The cross validation study of Eggstaff et al. (2014) (summarized in Cooke 2015) deserves particular mention in this regard. Using the 62 studies available at the inception of their research, Eggstaff et al. considered all splits of calibration variables into training sets and test sets. The performance weight *PW* model was initialized on each training set and evaluated on the complementary test set; corresponding equal weights *EW* solutions were also computed. Aggregating over all such splits, the ratios *PW*/*EW* shown in Fig. 4 speak for themselves.

The conclusion from these independent appraisals is that, while experts differ greatly in their ability to quantify uncertainty accurately and informatively, the superiority of performance-based combinations is amply attested, both in- and out-of-sample. Many researchers continue to eschew validation, and promote approaches known to have poor performance, such as quantile or harmonic averaging. The goal of science-based uncertainty quantification is not served by neglecting validation.

## Footnotes

1
http://rogermcooke.net/rogermcooke_files/Cross validation post 2006.pdf; http://rogermcooke.net/rogermcooke_files/Supplementary Material for Cross Validation.pdf

2
http://www.rogermcooke.net

3 These variables comprised the contributions to sea level rise from three accumulation/ablation processes, accumulation, run-off and discharge, for each ice sheet (East Antarctica is not shown on Fig. 3) for six different future scenarios: +2 °C, +3 °C, +4 °C global temperature rise by 2100 CE, +4 °C, +6 °C, +8 °C global temperature rise by 2200 CE. Which is which in Fig. 3 may be deciphered from the labels on the graphs.

## References

[CR1] Bamber JL, Aspinall WP (2013). An expert judgment assessment of future sea 1evel rise from the ice sheets. Nat Clim Chang.

[CR2] Cooke RM (1991). Experts in uncertainty; opinion and subjective probability in science.

[CR3] Cooke RM (2015). Messaging climate change uncertainty. Nat Clim Chang.

[CR4] Cooke RM, Wittmann ME, Lodge DM, Rothlisberger JD, Rutherford ES, Zhang H, Mason DM (2014). Out-of-sample validation for structured expert judgment of Asian carp establishment in Lake Erie. Integr Environ Assess Manag.

[CR5] Cooke RM, Goossens LLHJ (2008) TU Delft expert judgment data base. Reliab Eng Syst Saf 93:657–674

[CR6] de Vries H, van de Wal RSW (2015). How to interpret expert judgment assessments of twenty-first century sea-level rise. Clim Chang.

[CR7] Eggstaff JW, Mazzuchi TA, Sarkani S (2014). The effect of the number of seed variables on the performance of Cooke’s classical model. Reliab Eng Syst Saf.

[CR8] Lichtendahl KC, Grushka-Cockayne Y, Winkler RL (2013). Is it better to average probabilities or quantiles?. Manag Sci.

[CR9] Murphy JM, Sexton DMH, Barnett DN, Jones GS, Webb MJ, Collins M (2004). Quantification of modelling uncertainties in a large ensemble of climate change simulations. Nature.

[CR10] Vose Software (2013) http://www.vosesoftware.com/

